# Factor analysis of the relationship between PANSS score and family burden of patients with schizophrenia

**DOI:** 10.1002/brb3.2229

**Published:** 2021-06-14

**Authors:** Jiang Huang, Wei‐Xiang Wei, Pan‐Pan Zheng, Tao Tang, Pei‐Hao Zhang, Mao‐Yuan Long, Mei‐Ling Li, Xiao‐Yu Ning, Ying‐Yun Tian, Yu Cheng, Jun Wu

**Affiliations:** ^1^ Department of Psychiatry The Second Affiliated Hospital of Guizhou Medical University Kaili City China; ^2^ Guizhou Qiandongnan Ethnic Vocational and Technical College Kaili China; ^3^ Department of Chest and Breast, Qiandongnan People's Hospital

**Keywords:** disease, family burden, factor analysis, influence, positive and negative symptom rating scale, schizophrenia

## Abstract

**Objective:**

This study aims to investigate the burden of family caregivers of patients with schizophrenia, and its influencing factors

**Methods:**

A total of 105 patients with schizophrenia and their caregivers were investigated using the positive and negative symptom scale (PANSS) and family burden scale of disease (FBS)

**Results:**

There was a strong correlation between the patient's recovery and family burden, especially between positive and negative symptoms and family financial burden, family daily activities, family recreational activities, and family relationship

**Conclusion:**

There is a strong correlation between the patient's recovery and family burden, and this is especially correlated to family economic burden, family daily activities, family recreational activities, and family relationship. Medical staff should pay attention to the psychological characteristics of patients and fully understand and avoid the adverse effects of family burden on the rehabilitation of patients.

## Abstract 2

There is a strong correlation between the patient's recovery and family burden, and this is especially correlated to family economic burden, family daily activities, family recreational activities and family relationship. Medical staff should pay attention to the psychological characteristics of patients, and fully understand and avoid the adverse effects of family burden on the rehabilitation of patients.

## 1 INTRODUCTION

In today's social pressure, the number of schizophrenic patients has exhibited an increasing trend (Balter, 2017). In addition to the physiological and psychological factors of the patients themselves, social factors, especially family factors, play an important role in the onset, treatment, and rehabilitation of schizophrenia (Tandon et al., 2013). Many patients are taken care of by family members at home after discharge. Therefore, the family is very important for schizophrenic patients (Mantovani et al., 2016). However, schizophrenia also has a great impact on families, including the economic, social, and psychological aspects (Ribé et al., 2018). Therefore, the interaction between these two is very important for patients and families. The evaluation of the relationship between these two can provide great help to improve the status quo of patients and their families (Østergaard et al., 2016).

The positive and negative symptom scale (PANSS) is a standard that should be widely used in the clinical diagnosis of schizophrenia in psychiatry (Opler et al., 2017). PANSS is one of the most commonly used scales in psychiatry, which has good reliability and validity (Németh et al., 2018). It is suitable for the clinical diagnosis of schizophrenia, and has been widely used in China since 1990 (Hopkins et al., 2017). PANSS consists of four sub‐scales: positive scale, negative scale, general mental scale, and compound scale (Li et al., 2021). This scale is divided into seven grades of 1–7, in which 1 refers to asymptomatic and 7 refers to severe symptoms (Kay et al., 1987).

The family burden scale of disease (FBS) is a general questionnaire to comprehensively and systematically understand the family burden of patients (Hirsch et al., 2016). There are 24 items in six dimensions in the FBS, which include family economic burden (six items), family daily activities (fix items), family entertainment activities (four items), family relationship (five items), physical health of family members (two items), and mental health of family members (two items) (Frias et al., 2020). The scale adopts three grades of 0–2, in which no burden has a rating of 0 and severe burden has a rating of 2 (Frias et al., 2020). Since the number of items in each dimension is different, and in order to standardize the score, the score of each dimension was divided by the number of items to obtain the standardized average score and total average score (Liu et al., 2017). Then, the average score was divided into three grades, with a score of 0 indicating no burden and a score of 1 or 2 indicating a positive result (Stansfeld et al., 2017).

The hypothesis of this study is that there is a correlation between the patient's recovery and family burden. In order to prove it, the PANSS and FBS were used to survey 105 schizophrenic patients and their caregivers, and the factor analysis method of the multivariate statistics was used to construct a model for comprehensive evaluation. Finally, the burdens of the disease families were classified and ranked in order to determine the main problems of the burden of patient families.

## 2 STUDY METHODS

### 2.1 Inclusion criteria for subjects

The subjects were schizophrenic patients in the rehabilitation period. Patients must meet the ICD‐10 diagnostic criteria for schizophrenia (Castagnini & Fusar‐Poli, 2017). The course of the schizophrenia was ≥3 months, and the patient had no serious mental and physical illness during the accomplishment of the questionnaire. Inclusion criteria for patient caregivers: subjects within the age of 15–65 years, who are the children, spouses, or other family members of the patients, and are the main caregivers, legal guardians, or direct financial sources of the patients. Furthermore, the respondents should be able to fully understand the content of the scale and answer it normally.

### 2.2 Methods

From July 2018 to March 2019, a questionnaire survey was conducted by psychiatrists on 121 psychiatric patients and their families, who visited the Psychiatric Department of the Second Affiliated Hospital of Guizhou Medical University, Qiandongnan Prefecture, Guizhou Province during this period. The PANSS was filled in by the psychiatrist, according to the patient's symptoms, and the FBS was filled in by the patient's families when they visited. Finally, 105 valid questionnaires were collected, which involved 58 males, accounting for 55.2%, and 47 females, accounting for 44.8%. The average age of these subjects was 33.34 years (33.34 ± 11.09 years). In the distribution of educational background, six subjects were from college for professional training graduates, accounting for 6.7%, while 16 subjects were high school and technical secondary school graduates, accounting for 15.2%. Furthermore, 78 subjects came from rural areas, accounting for 74.3%, while 27 subjects came from towns and cities, accounting for 25.7%. Moreover, 82 subjects had middle school or primary school educational levels, or were illiterates, accounting for 78.1%.

### 2.3 Statistical data processing

The data were statistically analyzed using the statistical software SPSS 17.0 and using *t*‐test or factor analysis.

## 3 RESULTS

### 3.1 General situations of the PANSS and FBS scores

The absolute value of the correlation coefficient between the total PANSS and FBS scores was 0.604 (*p *< .01). This indicates that these two are moderately correlated.

In Table 1, the results in the correlation coefficient table reveal that the absolute value of the correlation coefficient between the total PANSS and FBS scores was 0.619 (*p *< .01). This indicates that these two are strongly correlated. The absolute value of the correlation coefficient between the total scores of the PANSS and family daily activities was 0.586 (*p *< .01). This indicates that these two are moderately correlated. The absolute value of the correlation coefficient between the total scores of the PANSS and family entertainment activities was 0.566 (*p *< .01). This indicates that these two are moderately correlated. The absolute value of the correlation coefficient between the total scores of the PANSS and family relationship was 0.458 (*p *< .01). This indicates that these two are moderately correlated. The absolute value of the correlation coefficient between the total scores of the PANSS and the physical health of family members was 0.149 (*p *> .05). This indicates that these two are weakly correlated. The absolute value of the correlation coefficient between the total scores of the PANSS and the mental health of family members was 0.227 (*p *< .05). This indicates that these two are weakly correlated.

**Table 1:** Significance test of positive and negative syndrome scores and family burden scale

	Family financial burden	Daily family activities	Family entertainment	Family Relations	Family members are physically healthy	Mental health of family members
Positive and negative symptom scale scores	.619*	.586*	.566*	.458*	.149	.227*

* *p* < 0.05; **: *p* < 0.01.

The above analysis reveals that the total PANSS and FBS scores were moderately positively correlated. This indicates that when the number of positive and negative symptoms increases, the burden of the patient's family would also increase.

In Table 2, the results of the questionnaire survey revealed that the family burden of the patient's family members are as follows: among the six items, family economic burden has the highest positive rate (60 positive responses), accounting for 57.14%, family daily activities had 48 positive responses, accounting for 45.71%, the mental health of the family members had the lowest positive rate (12 positive responses), accounting for 11.43%, and the total burden of the family had 30 positive responses, accounting for 28.57. The standardized score of the total average score of burden was 17.40 ± 10.87.

**Table 2:** Scores of positive and negative syndromes and general scores of family burden scale

	Mean value	Standard deviation	Number of positive answers	A positive answer)%(
Positive and negative symptom scale scores	60.30	34.525	–	–
Family burden of disease scale score	17.40	10.876	30	28.57
Family financial burden	5.79	2.942	60	57.14
Daily family activities	3.54	2.759	48	45.71
Family entertainment	3.10	2.510	48	45.71
Family Relations	3.96	2.862	49	46.67
Family members are physically healthy	.57	.943	16	15.24
Mental health of family members	.45	.880	12	11.43

### 3.2 Factor analysis of the FBS

Six indexes were selected for the factor analysis, and the specific indexes were as follows: economic burden X1, family daily activities X2, family entertainment activities X3, family relationship X4, family member physical health X5, and family member mental health X6.

From the correlation coefficient matrix above, it is concluded that most of the variables were greater than 0.3. This indicates that the selected data is suitable for the factor analysis.

The results exported by the SPSS software are shown in Table 3. It can be observed that the value of KMO is 0.829. This indicates that the effect of the factor analysis was excellent. Furthermore, the Bartlett's sphericity test revealed a *p*‐value of < .001. Therefore, this was suitable for the factor analysis.

**Table 3:** KMO and Bartlett tests

The Kaiser–Meyer–Olkin measure with sufficient sampling degree		.829
Bartlett's test of sphericity	Bartlett's test of sphericity	371.797
	*df*	15
	Sig.	.000

### 3.3 Extraction of common factors and determination of the cumulative variance contribution rate

The common factors were extracted by principal component analysis. In general, the extracted common factor eigenvalue is greater than 1. However, sometimes, in order to explain the index variables more accurately, factors with eigenvalues of less than 1 can also be extracted. In the present study, some common factors with eigenvalues greater than 0.8 were extracted. In order to verify the degree of information loss of the original variables, the commonality of variables was calculated:

$$\begin{array}{c} h_{i}^{2}=a_{i1}^{2}+a_{i2}^{2}\\ h_{1}^{2}=0.876^{2}+{\left(-0.261\right)}^{2}\\ h_{2}^{2}=0.871^{2}+{\left(-0.200\right)}^{2}\\ \vdots \\ h_{6}^{2}=0.628^{2}+0.643^{2} \end{array}$$

That is, after being extracted from the two common factors, the commonalities of variables were almost above 80% (the detailed results are presented in Table 1). This indicates that the extracted common factors already contain most of the information of the original variables, and lost information was relatively less. Therefore, the effect of the factor analysis was relatively ideal.

Eigenvalues can also be considered as variance contributions:

$$g_{j}^{2}=a_{1j}^{2}+a_{2j}^{2}+\cdots +a_{pj}^{2}\;\left(j=1,2,\ldots ,m\right).$$

That is,

$$\begin{array}{c} g_{1}^{2}=0.876^{2}+0.871^{2}+0.856^{2}+0.821^{2}+0.685^{2}+0.648^{2}\\ g_{2}^{2}={\left(-0.261\right)}^{2}+{\left(-0.200\right)}^{2}+{\left(-0.284\right)}^{2}+{\left(-0.203\right)}^{2}+0.577^{2}\\ \;+\;0.643^{2} \end{array}$$

$$\mathrm{Variance}\;contribution\;rate=\frac{g_{j}^{2}}{p}\times 100\%.$$

Then

$$\begin{array}{c} \mathrm{Variance}contributionrateofthefirstgroupofcomponents\\ =\frac{3.823}{6}\times 100\%=63.718\%; \end{array}$$

$$\begin{array}{c} \mathrm{Variance}contributionrateofthesecondgroupofcomponents\\ =\frac{0.977}{6}\times 100\%=16,285\%. \end{array}$$

**FIGURE 1:**
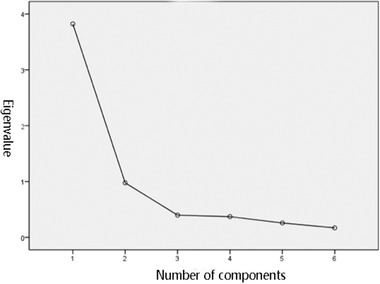
Gravel map

The cumulative variance contribution rate of the three common factors extracted was 63.718% + 16.285% = 80.003%. This indicates that it is feasible to extract two common factors. On the basis of the gravel map in Figure 1, it can be observed that through the third factor, the eigenvalue began to stabilize. Therefore, it would be most appropriate to extract two common factors.

### 3.4 Establishment of the factor model

In order to make the meaning of the common factor easier to explain, factor rotation was required to be conducted. In the present study, the initial factor load matrix was rotated using the orthogonal rotation method, in which the initial load matrix was multiplied to the orthogonal matrix on the right. The initial factor load matrix was rotated using the SPSS software. According to the factor score coefficient matrix, the expression of the factor score can be obtained as follows:

$$F_{j}=\beta_{j1}X_{1}+\beta_{j2}X_{2}+\cdots +\beta_{jp}X_{p},Xj=1,2,\ldots ,m.$$

That is,

$$\begin{array}{c} F_{1}=0.291X_{1}+0.343X_{2}+0.335X_{3}+0.302X_{4}+\left(-0.156\right)X_{5}\\ \;\;+\;\left(-0.200\right)\;X_{6}\\ F_{2}=\left(-0.065\right)X_{1}+\left(-0.131\right)X_{2}+\left(-0.108\right)X_{3}\\ \;\;+\left(-0.056\right)X_{4}+0.597X_{5}+0.650X_{6} \end{array}$$

where $X_{i}\;\left(i=1,2,\ldots ,19\right)$ represents the data after standardization. Finally, the comprehensive score was calculated, with the proportions of the variance contribution rate of each factor in the cumulative contribution rate taken as the weight for the weighted summary. Afterwards, the comprehensive score of each area was calculated. That is,

$$F=\frac{\left(63.718F_{1}+16.285F_{2}\right)}{80.003}$$

Then, the scores of each factor and comprehensive scores were ranked. The results are presented in Table 4.

**Table 4:** Ranking of scores of each factor and comprehensive scores

No.	F1	F1 ranking	F2	F2 ranking	F	F ranking
1	0.71955	21	1.09528	13	0.8	15
2	0.71955	21	1.09528	13	0.8	15
3	0.46351	32	−0.19259	40	0.33	31
4	0.43855	35	1.78641	10	0.71	19
5	−1.42385	99	−0.25784	48	−1.19	99
6	0.70827	23	−0.77746	90	0.41	27
7	2.67843	1	−0.72501	86	1.99	2
8	2.01614	5	1.809	9	1.97	4
9	−0.68339	75	−0.42757	68	−0.63	77
10	−0.2836	60	−0.51493	76	−0.33	65
11	−0.46001	69	−0.49694	74	−0.47	73
12	−1.2446	94	−0.27388	51	−1.05	94
13	−1.15178	89	−0.2984	56	−0.98	90
14	−0.67073	74	−0.45804	71	−0.63	77
15	−1.15178	89	−0.2984	56	−0.98	90
16	−1.00381	85	2.99612	3	−0.19	60
17	−0.24438	59	0.67021	17	−0.06	53
18	0.42637	36	−0.70447	85	0.2	45
19	−1.34996	97	−0.25443	45	−1.13	97
20	−0.44748	66	−0.49186	72	−0.46	71
21	−0.59008	71	2.02659	6	−0.06	53
22	−0.60274	72	2.05707	5	−0.06	53
23	−0.43198	65	−0.52038	77	−0.45	70
24	0.41837	37	−0.7872	91	0.17	47
25	−0.78248	79	−0.40558	65	−0.71	80

## 4 DISCUSSION

The present study result confirmed our hypothesis that the rehabilitation factors of patients are moderately correlated to the economic burdens of their families, and that it is possible to provide better social support for patients by popularizing family mental health knowledge. Furthermore, the factor analysis method of the multivariate statistical analysis was used to construct the corresponding model, and comprehensively evaluate the family burdens of 105 patients. The SPSS software was used to process the related data, and the processing results were analyzed. Finally, the family burdens of 105 patients were classified and ranked, in order to provide a basis for putting forward the corresponding suggestions on the main problems that exist in the family burdens of patients. Table 4 clearly shows the different family burdens of each patient. This also reflects the characteristics of the different burdens of the patient's disease on the family (DeTore et al., 2018; Leguay, 2016; Yu et al., 2017). With a reasonable explanation for each common factor, and combined with the score of each common factor and the comprehensive scores on the family burdens of each patient, the family burdens of each patient can be simply evaluated. According to the severity of family burdens of patients in different periods, focus should be given on the diagnoses of the different conditions of patients.

For instance, the 7th patient ranked first on the common factor F1, 86th on F2, and 2nd on the comprehensive score *F*. This indicates that the family of the 7th patient had heavy burdens in terms of economic concern, daily activities, entertainment activities and family relationship. However, they did not have very heavy burdens in terms of physical health and mental health. In general, the family burden of the 7th patient was heavy. The 97th patient ranked 16th on the common factor F1, 103rd on F2 and 20th on the comprehensive score *F*. This indicates that the family of the 97th patient had heavy burdens in terms of economic concern, daily activities, entertainment activities, and family relationship. However, they had relatively light burdens in terms of physical health and mental health. In general, the family burden of the 97th patient was relatively heavier. The 98th patient ranked 103rd on the common factor F1, 38th on F2 and 103rd on the comprehensive score *F*. This indicates that the family of the 98th patient had light burdens in terms of economic concern, daily activities, entertainment activities, and family relationship. However, they had relatively heavy burdens in terms of physical health and mental health. In general, the family burden of the 98th patient was not light.

According to the results of the analysis, the problems and difficulties of family burdens of different patients can be determined, and reasonable and feasible policy recommendations can be put forward. Therefore, it is of great significance to analyze and investigate the family burdens of the families of patients. Viewing as a whole, family economic burden, daily activities, entertainment activities, and family relationship accounts for a large proportion of the burdens of families of patients. In order to improve the family burdens of patients, this should be initiated on the economic burdens, daily activities, entertainment activities and family relationship of the patient's families.

The novelty of our study is that, according to the results of the present study, the following new ideas are provided for the rehabilitation of schizophrenic patients in a hospital. First, for patients with heavy economic burdens in their families, the corresponding subsidies and economic subsidies can be obtained from local civil affairs departments. If the patient returns to the hospital again, attempts should be made to save the patient's family economic and medical expenses. Second, for the patients who are about to leave the hospital in the rehabilitation stage, the hospital nursing staff should focus their attention in carrying out some family daily activities and recreational activities in order to help patients integrate into the society faster. For patients who have already recovered, the corresponding guidance should be given to the patient's families. The patient's families should be instructed to provide the corresponding help, and the families should focus their attention to the patient's participation in the daily activities of the family. Finally, the patient's family should focus their attention in improving the family relationship and creating a good family atmosphere, and especially allow the patient to feel a harmonious family interpersonal relationship, in order to improve the mental symptoms of the patients (Caqueo‐Urízar et al., 2015; Koutra et al., 2014). However our limitation is that the sample size is not large enough, thus further study is needed.

## 5 CONCLUSION

There is a strong correlation between rehabilitation and family burden. In particular, family economic burden is correlated to family daily activities, family entertainment activities, and family relationship. The medical staff should focus their attention to the psychological characteristics of these patients and fully recognize and avoid the adverse effects of family burden during the patient's rehabilitation.

## FUNDING INFORMATION

The work was supported by Guizhou Qiandongnan Science and Technology Plan Project, Grant Number: Qiandongnan Kehe J [2018] 049 (NJD)

### PEER REVIEW

The peer review history for this article is available at https://publons.com/publon/10.1002/brb3.2229.

## Data Availability

The datasets used and/or analyzed during the current study available from the corresponding author on reasonable request.
